# Melatonin improves cognitive dysfunction and decreases gliosis in the streptozotocin-induced rat model of sporadic Alzheimer’s disease

**DOI:** 10.3389/fphar.2024.1447757

**Published:** 2024-07-29

**Authors:** Zsolt Gáll, Bernadett Boros, Krisztina Kelemen, Melinda Urkon, István Zolcseak, Kincső Márton, Melinda Kolcsar

**Affiliations:** ^1^ Department of Pharmacology and Clinical Pharmacy, George Emil Palade University of Medicine, Pharmacy, Science, and Technology of Targu Mures, Târgu Mures, Romania; ^2^ Faculty of Pharmacy, George Emil Palade University of Medicine, Pharmacy, Science, and Technology of Targu Mures, Târgu Mures, Romania; ^3^ Department of Physiology, George Emil Palade University of Medicine, Pharmacy, Science, and Technology of Targu Mures, Târgu Mures, Romania; ^4^ Faculty of Medicine, George Emil Palade University of Medicine, Pharmacy, Science, and Technology of Targu Mures, Târgu Mures, Romania

**Keywords:** melatonin, Alzheimer’s disease, animal model, cognitive dysfunction, neuroinflammation

## Abstract

**Introduction:**

Alzheimer’s disease (AD) and other forms of dementia have a devastating effect on the community and healthcare system, as neurodegenerative diseases are causing disability and dependency in older population. Pharmacological treatment options are limited to symptomatic alleviation of cholinergic deficit and accelerated clearance of β-amyloid aggregates, but accessible disease-modifying interventions are needed especially in the early phase of AD. Melatonin was previously demonstrated to improve cognitive function in clinical setting and experimental studies also.

**Methods:**

In this study, the influence of melatonin supplementation was studied on behavioral parameters and morphological aspects of the hippocampus and amygdala of rats. Streptozotocin (STZ) was injected intracerebroventricularly to induce AD-like symptoms in male adult Wistar rats (n = 18) which were compared to age-matched, sham-operated animals (n = 16). Melatonin was administered once daily in a dose of 20 mg/kg body weight by oral route. Behavioral analysis included open-field, novel object recognition, and radial-arm maze tests. TNF-α and MMP-9 levels were determined from blood samples to assess the anti-inflammatory and neuroprotective effects of melatonin. Immunohistological staining of brain sections was performed using anti-NeuN, anti-IBA-1, and anti-GFAP primary antibodies to evaluate the cellular reorganization of hippocampus.

**Results and Discussion:**

The results show that after 40 days of treatment, melatonin improved the cognitive performance of STZ-induced rats and reduced the activation of microglia in both CA1 and CA3 regions of the hippocampus. STZ-injected animals had higher levels of GFAP-labeled astrocytes in the CA1 region, but melatonin treatment reduced this to that of the control group. In conclusion, melatonin may be a potential therapeutic option for treating AD-like cognitive decline and neuroinflammation.

## 1 Introduction

Alzheimer’s disease (AD) is an age-related neurodegenerative pathology, the most common type of dementia which affects around 50 million people worldwide and the exact cause remains unknown (Livingston et al., 2017). Several hypotheses attempt to explain the main features of AD, such as progressive cholinergic decline, β-amyloid accumulation, and a series of metabolic alterations leading to neurofibrillary tangle formation and insulin resistance in the brain (Estrada and Contreras, 2019; Kang et al., 2020). Most drugs developed to reduce amyloid synthesis failed to show clinically relevant improvement and despite the vast amount of resources invested in development and research only two new treatment options have been registered for AD patients in the last decade (Decourt et al., 2022; Cummings et al., 2024). Overall, there is an obvious β-amyloid peptide accumulation in the nerve cells that leads to early axonopathy, synaptic loss, neuronal destruction (Rosales-Corral et al., 2012) and the phenomenon predominantly affects the signaling pathways of memory-related molecular signatures (Kaushik et al., 2022). Hippocampal dysfunction is a commonly observed feature of Alzheimer’s disease, which can involve metabolic disturbances (i.e., hypometabolism) and morphological reorganization (Small et al., 2011; Klyubin et al., 2012; Padurariu et al., 2012). The main symptom (i.e., memory disturbances) is primarily caused by the destruction of cholinergic neurons, but β-amyloid accumulation and oxidative stress can also reduce acetylcholine synthesis by reducing choline acetyl transferase activity or increasing acetylcholinesterase (AChE) activity (Rosales-Corral et al., 2012).

Due to the progressive character of AD, prevention and early diagnosis can be effective options in the symptomatic treatment of the disease. Several risk factors, such as systemic inflammation (i.e., autoimmune diseases), obesity, diabetes, and hypertension were identified but effective and tolerable interventions are sparse. Even though neuronal damage characterized by the accumulation of toxic proteins, inflammation, and atrophy can be slowed down by certain drugs, the efficacy of immune-modulatory, anti-inflammatory, or neuroprotective agents depends on early diagnosis (Munafò et al., 2020; Pais et al., 2020). As a recent meta-analysis demonstrated, regular physical activity was the only protective factor against dementia and Alzheimer’s disease that all adults could benefit from (Iso-Markku et al., 2022). It has also been shown that sleep disorders can cause cognitive impairment, and impaired melatonin synthesis has been found in Alzheimer’s patients (Cardinali D et al., 2010). In addition, melatonin secretion might increase after moderate-intensity exercises (Kaylee et al., 2020). Moreover, longer sleep duration leads to reduced β-amyloid levels in older cognitively unimpaired adults (Insel et al., 2021). Consequently, melatonin might be the key mediator of the beneficial effects of sleep on the clearance of β-amyloid from the brain (Li et al., 2020).

Melatonin (N-acetyl-5-methoxytryptamine) is the primary secretory product of the pineal gland, but it is also produced in the gastrointestinal tract, respiratory epithelium, pancreas, adrenal glands, thyroid gland, thymus, urogenital tract, placenta and even in non-endocrine cells (Kvetnoy et al., 2022). Indeed, it regulates biorhythm and controls sleep, but at molecular levels it is involved in the metabolism of free radicals due to its antioxidant capacity. It modulates immune responses, cell proliferation and differentiation, and also has neuroprotective effects in several preclinical models (Rosales-Corral et al., 2012; Miranda-Riestra et al., 2022; Tozihi et al., 2023). On the other hand, decreased levels of melatonin were observed in the cerebrospinal fluid of elderly subjects with early AD-like neuropathological changes (Rosales-Corral et al., 2012). Interestingly, pinealectomy causes pyramidal cell loss in the cornu Ammonis 1 (CA1) and cornu Ammonis 3 (CA3) regions of rats, which can be reversed by melatonin supplementation (De Butte and Pappas, 2007).

This study aimed to investigate whether melatonin treatment would reverse the neuroinflammation and cellular reorganization in the sporadic model of Alzheimer’s disease in rats induced by intracerebroventricular (ICV) injection of streptozotocine (STZ). Behavioral, biochemical, and histopathological tests were conducted to assess the effect of subchronic melatonin treatment on cognitive performance and neuronal damage, respectively.

## 2 Materials and methods

### 2.1 Drugs and administration

STZ was purchased from Cayman Chemicals. Rats in the STZ group received an ICV injection of STZ (3 mg/kg body weight) dissolved in 8 μL citrate buffer (0.05 M, pH = 4.5), and the rats in the control (CTRL) group received an equal volume of vehicle. Melatonin (Cayman Chemicals, Ann Arbor, MI, United States) was dissolved in ethyl alcohol (50 mg/mL) and incorporated in food pellets, followed by solvent evaporation. The pellets were freshly prepared before drug administration, and the amount of melatonin was calculated for each animal using a 20 mg/kg body weight dose per day.

### 2.2 Animals and experimental groups

The experiment was carried out on 5–6-month-old male Wistar rats (350–450 g). They were kept in a polypropylene cage (1291H Eurostandard Type III H, 425 mm × 266 mm × 185 mm, Techniplast, Milan, Italy) and maintained under standard housing conditions (temperature 20°C ± 2°C and humidity 60% ± 10%) with a 12-h light-dark cycle. Animals were housed singly 7 days prior to the start of the experiments. No environmental enrichment, including hiding spaces, was provided. Dry food pellets (Cantacuzino Institute, Bucharest, Romania) and water were available *ad libitum*. The rats were procured from the Biobase of UMFST George Emil Palade of Targu Mures, Romania. The experimental protocol was approved by the Ethics Committee of George Emil Palade University of Medicine, Pharmacy, Science, and Technology of Targu Mures (approval no. 366/2018) and the National Authority (no. 123/2018).

Thirty-four rats were randomly divided into four groups, from which two groups consisted of sham-operated animals (CTRL and CTRL + MEL) and the other two received ICV-STZ injections (STZ and STZ + MEL). The first group of animals (CTRL) was injected with citrate buffer ICV and administered orally with pellets containing vehicle used for melatonin dosing (n = 8); the second group (CTRL + MEL) was injected with citrate buffer ICV and administered with pellets containing melatonin (n = 8); the third group (STZ) was injected ICV with STZ and received vehicle containing pellets (n = 9); the fourth group (STZ + MEL) was injected ICV with STZ and received melatonin containing pellets (n = 9). Melatonin (20 mg/kg) was administered daily, beginning on the sixth day of the experiment until the end of the study. Total treatment duration was 30 days, which corresponds to a subchronic treatment regimen.

Briefly, animals were anesthetized with a mixture of ketamine (100 mg/kg body weight) and xylazine (10 mg/kg body weight) through intraperitoneal administration. Rats were then placed into a stereotactic apparatus (Digital Stereotaxic with Manual Fine Drive, Leica Biosystems, Buffalo Grove, IL, United States) and injected bilaterally with STZ at a sub-diabetogenic total dose of 3 mg/kg (dissolved in 0.05 M citrate buffer, pH 4.5, in one single dose on day one) using a 10 μL Hamilton syringe (Hamilton^®^ glass syringe 700 series RN, Hamilton syringe Hamilton syringe (Hamilton^®^ glass syringe 700 series RN), Hamilton, Bonaduz, Switzerland) with a 26-G needle into each ventricle (4 µL/ventricle). The sham-operated animals received an equal volume of vehicle (0.05 M citrate buffer, pH 4.5) by the same procedure. Stereotactic coordinates relative to bregma, dura mater and inter-hemispheric scissure: antero-posterior: −0.7 mm; ventral: −3.6 mm; lateral: ±1.5 mm. The injection speed was set to 0.4 μL/min using a motorized programmable stereotaxic injector (Stoelting QSI Model 5311, Stoelting Co., Wood Dale, IL, United States), and the injection needle remained for at least 2 min in place, to prevent backflow of the administered solution (Kraska et al., 2012; Knezovic et al., 2015; Homolak et al., 2021). Postoperatively, animals were hydrated intraperitoneally with 6 mL saline solution and monitored for signs of pain.

After the ICV injection, animals were allowed a 5-day recovery period during which they were kept in individual home cages and their body weight, food, and water intake were monitored daily. Although STZ exhibit specific pancreatic β-cell toxicity when injected intraperitoneally, the STZ-ICV injection did not affect peripheral blood glucose levels and body weight as demonstrated previously (Song et al., 2018). Immediately after recovery, all animals were randomly assigned to a control or treatment group. Due to mortality observed in the STZ-injected groups (5 animals died shortly after ICV injection) that could be associated with STZ acute toxicity as described earlier (Chen et al., 2014; Guardia de Souza e Silva et al., 2020; Gáspár et al., 2022), 29 animals were included in the behavioral, biochemical, and histological analysis. The number of animals/group at the end of the experiment was as follows: CTRL n = 8, CTRL + MEL n = 8, STZ n = 7, STZ + MEL n = 6. In exceptional situations, where biological samples obtained were not validated due to sampling procedure errors, the number of samples used for those analyses was specified.

### 2.3 Behavioral studies

**FIGURE 1:**
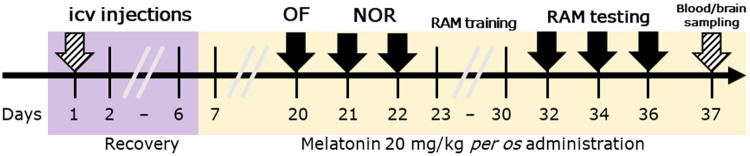
Timeline of the study design. Legend: OF, open field test; NOR, novel object recognition test; RAM, radial arm maze test; ICV, intracerebroventricular. Solid arrows represent behavioral tests, while striped arrows represent surgical interventions.

On the 20th day of the experiment counted from the day of STZ injection, behavioral testing began with an open field test, followed by novel object recognition and radial arm maze tests. The timeline of the experiment is shown in Figure 1.

#### 2.3.1 Open field test

To assess the exploratory and locomotor activity of the animals, the first behavioral test implemented was an open field test on day 20. The open field apparatus consisted of a black floor (60 × 60 cm) and transparent walls (60 cm). The intensity of the illumination was controlled for and set to an average light intensity of 150 ± 10 lux. The rats were placed in the center of the box and left undisturbed to explore the apparatus for 5 min. The activity of rats was monitored and recorded with a CMOS video camera (30 fps) and behavior analysis software (Ethovision XT, version 11.5, Noldus IT, Wageningen, Netherlands). The detection options were optimized for each trial. Automatic analysis was used to detect the rat at three points (head, body, tail) and record the distance moved, the number of entries and the time spent in different zones of the apparatus. In addition, rearing and grooming were measured by semi-automatic analysis performed by two blinded observers.

#### 2.3.2 Novel object recognition test

There is a wide range of uses for the NOR test, including the assessment of memory deficits, anxiety, attention, novel object preferences, exploratory patterns, and pharmacological therapy (Antunes and Biala, 2012). The apparatus consisted of an open box made of Plexiglas (60 × 60 cm) and transparent walls (60 cm) positioned on the floor. The light intensity was set to 80 ± 10 lux.

The objects to be explored were made of plastic, glass, or wood. The height of the objects was approximately the same (5 ± 2 cm), and they were fixed to the base of the apparatus to prevent displacement by animals. Objects were positioned 10 cm away from the box walls, allowing animals to move along the walls undisturbed. After each trial, 70% alcohol was used to clean the box and the objects, removing olfactory cues. The familiar and novel objects were counterbalanced in the left and right positions, to prevent bias towards a particular location. Object exploration was quantified by Ethovision XT 11.5 software using the nose point of the rats (touching or no more than 0.5 cm away from the object) but activities such as leaning against, turning around, standing, or sitting on the objects were excluded.

Two inter-trial intervals were used to determine the short- or long-term memory of the rats, and all rats were subjected to three trials: one acquisition trial and two retention trials. The first was the acquisition trial, performed using two identical wood cuboid objects. Second, an intertrial period of 2 h was followed by the first retention trial, where one familiar object was replaced by a cylindrical transparent glass. The third trial was another retention trial completed after a 24-h inter-trial period. In this trial, the previously novel object was replaced with a black, round plastic object. In every phase, the animals had 5 min to explore the environment and the objects (Mathiasen and DiCamillo, 2010). Consequently, short-term memory was evaluated 2 h after the presentation of identical objects, while long-term memory was assessed 24 h after the acquisition trial. The main parameter of this test was the discrimination index (DI), calculated using the time spent with the novel (TN) and the familiar object (TF) as Eq. 1 shows:

DI=(TN − TF) / (TN+TF) (1)

The DI is a number between −1 and +1. If it is near −1, the animal prefers the familiar object, if it is near +1, they prefer the novel one.

#### 2.3.3 Radial arm maze test

The cognitive functions of the animals, such as spatial learning and memory, were tested with the radial arm maze test (RAM). The RAM test is an 8-arm radial maze used to observe visuospatial learning ability and memory. The apparatus used for testing was initially described by Olton and Samuelson (1976) and was constructed in-house according to standard specifications: an octagonal center platform surrounded by eight wooden arms (50 cm long and 10 cm wide) with 20 cm high walls; at the end of each arm there was a hole, which contained food that was not visible from the central platform. To test both working and reference memory of rats, two out of the eight arms did not contain food pellets (the unbaited arm). The position of the baited and unbaited arms was the same across all trials. Various environmental marks and objects (pictures, doors, tables, windows) were available to assist with spatial orientation.

After 2 days of acclimatization to testing conditions (apparatus, laboratory conditions with controlled visual cues) and to food restriction (5 days of food deprivation until reaching 80%–85% of their *ad libitum* body weight) to ensure their motivation for task completion, animals underwent a training phase that comprised one daily test and lasted for 7 days. The diet restriction was necessary to motivate the animals to explore the labyrinth.

The test phase was comprised of four trials conducted every other day. All trials were terminated after 10 min or after all pellets were found (Fole et al., 2017). Each animal was placed on the center platform of the apparatus in random order and their activity was observed and recorded with a camera (CMOS, 30 frame/sec) mounted above the apparatus. Researchers observed the experiment in the next room. The behavioral parameters were recorded offline using Ethovision XT software (Noldus IT, Netherlands). The following parameters were automatically measured: the number of baits collected or target visits, the total number of errors, which included the reentry into a previously visited baited or unbaited arm (working memory error), the first entry into an unbaited arm (reference memory error). To standardize the olfactory background, a disinfectant solution containing ethanol and isopropanol was used for cleaning before the first and after each test.

### 2.4 Biochemistry

Based on serum samples obtained by cardiac puncture and followed by centrifugation, TNF-α and MMP-9 levels were determined using commercial test kits (St John’s Laboratory, London, United Kingdom, catalog numbers STJ150174 and STJ150078, respectively). Quantitation was performed using optical density measurement on Luminex 200 instrument (Luminex Corp., Austin, TX, United States). The results were expressed as ng/mL.

### 2.5 Immunohistochemical procedures

#### 2.5.1 Sample collection

Rats were transcardially perfused with ice-cold saline for 1.5 min followed by an ice-cold fixative solution containing 4% paraformaldehyde (Sigma Aldrich, St. Louis, MO, United States) and 0.25% picric acid (Sigma Aldrich, St. Louis, MO, United States) in phosphate buffer (pH = 7.4) for 20 min under anesthesia with ketamine hydrochloride (100 mg/kg) and xylazine (10 mg/kg) injected intraperitoneally on the 37th day after STZ injection. We collected blood and brain samples. Blood was collected via cardiac puncture before perfusion. After perfusion, the brain was carefully removed, placed in fixative solution and after 48 h, placed into 0.1 M phosphate buffer solution. 60 μm-thick coronal sections were cut with a microtome (VT 1000S, Leica, Nussloch, Germany) and stored at 4°C until immunohistochemistry was performed.

#### 2.5.2 Fluorescent immunohistochemistry

Triple immunofluorescent staining was used to identify neurons, astrocytes, and microglia (Gáll et al., 2022). Sections were washed in 0.1 M phosphate buffer (PB, pH 7.4, Sigma Aldrich, St. Louis, MO, United States) and transferred to a 24-well tissue culture plate (TPP, Trasadingen, Switzerland). The plates were placed on an orbital shaker (Heidolph, Germany) and incubated at 37°C for 10 min for antigen retrieval. All samples were stained in a free-floating manner in 24-well tissue culture plates in 500 μL volume on an orbital shaker. After being washed 3 times for 10 min in 0.1 M PB, sections were washed in tris buffered-saline (TBS) (3 times 10 min), then incubated in a blocking solution of TBS, containing 10% normal goat serum (NGS) (Vectro Laboratories, Inc., Burlingame CA, United States, 94010), followed by incubation with a mixture of primary guinea pig polyclonal anti-NeuN antibody (guinea pig raised-polyclonal, dilution 1:500; product no: 266004, Synaptics Systems GmbH, Goettingen, Germany), primary rabbit polyclonal anti-IBA-1 antibody (rabbit raised-polyclonal, dilution 1:500; product no: HS234013, Synaptics Systems GmbH, Goettingen, Germany), and primary mouse monoclonal anti-GFAP antibody (mouse raised-monoclonal dilution 1:500; product no: 173211, Synaptics Systems GmbH, Goettingen, Germany) diluted in TBS containing 2% NGS and 0.1% Triton X-100 (Sigma Aldrich) for 24 h at room temperature. Sections were washed three times in TBS, then secondary antibodies made up in TBS, containing 2% NGS at room temperature for 2 h were applied to label the NeuN immunostaining with Alexa488-conjugated donkey anti-guinea pig (1:500, Jackson ImmunoResearch Laboratories, West Grove, PA, United States), the IBA1 immunostaining with Alexa594-conjugated donkey anti-rabbit (1:500, Jackson ImmunoResearch Laboratories, West Grove, PA, United States), and the GFAP immunostaining with Alexa647-conjugated donkey anti-mouse (1:500, Jackson ImmunoResearch Laboratories, West Grove, PA, United States). The stained sections were washed 3 × 10 min in TBS followed by 3 × 5 min in 0.1 PB, then mounted on slides and coverslipped with mounting medium containing 4′,6-diamidino-2-phenylindole (DAPI) for cell nuclei labeling (Vectashield with DAPI, Vector Laboratories, Burlingame, CA, United States) and sealed with nail polish. A negative control was prepared for all immunostaining by omitting the primary antibodies.

The sections were analyzed using 3DHISTECH Pannoramic MIDI II slide scanner collaborating with the Institute of Experimental Medicine (Budapest, Hungary). Fluorescent images were obtained with Zeiss, Plan-Apochromat ×20 dry objectives, Cy3.5 SB, Cy5-Q, LED-FITC and DAPI-Q filters. Digitized images were processed with ImageJ Fiji (v.1.5.3t with Java 1.8.0_322) and Ilastik (v.1.4.0) softwares. In the first step, the supervised machine learning process was facilitated by manually annotating the signal and the background pixels. Then, the pixel classification workflow was implemented as described by Berg et al. (2019). The resulting images were used for quantitative analysis. The total cell count and percent labelled area were determined using the “analyze particles” command that counts and measures objects in threshold images in ImageJ Fiji. Hippocampal regions and layers were differentiated based on cell density and relative location using DAPI staining. CA1 and CA3 regions of the hippocampus were quantified individually in four hippocampal sections per animal. The final value of each measurement presented is its group average (± standard deviation, SD).

### 2.6 Statistical analysis

All data was checked for normal distribution and was treated accordingly. The normality of raw data was ruled by Kolmogorov-Smirnov test using GraphPad Prism eight software (GraphPad Software, United States), the same software being used for figure generation. Data with a normal distribution were presented as mean ± SEM. A two-way ANOVA analysis was employed to examine the main effects of ICV-STZ injection and melatonin treatment. In the case of some parameters where repeated measurements were performed (NOR and RAM tests) a three-way ANOVA analysis was used. The *post hoc* Tukey’s test was then performed for multiple comparisons. Where the sample sizes were different, Sidak’s multiple comparison test was implemented. Nonparametric distributed data were analyzed by a Kruskal-Wallis test followed by Dunn’s *post hoc* test to detect the significant differences between groups *p* < 0.05 was considered statistically significant.

## 3 Results

### 3.1 Effects of melatonin supplementation on behavior

#### 3.1.1 Open-field test

**FIGURE 2:**
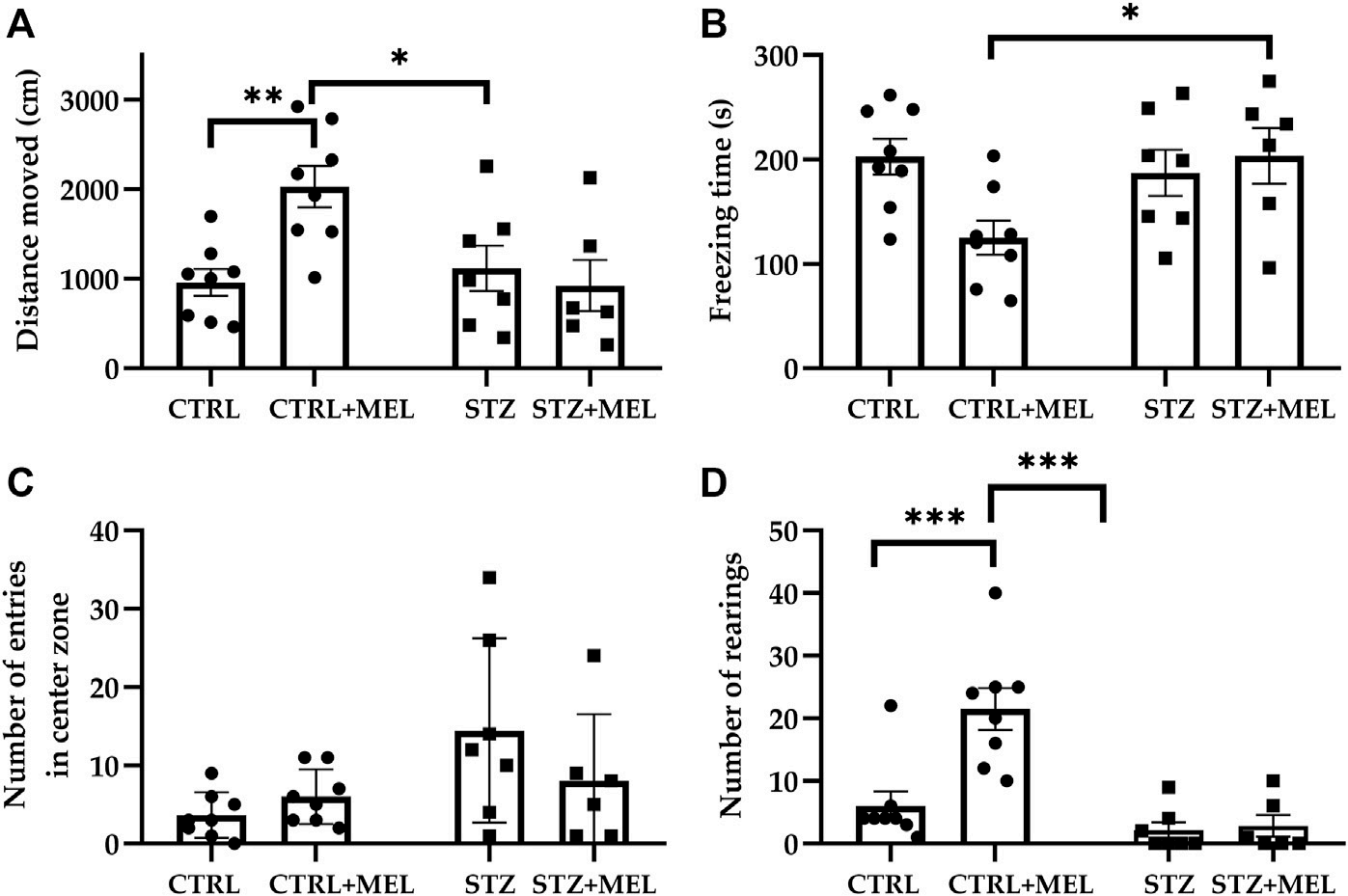
Open field test performance of melatonin treated rats underwent ICV-STZ injection. Quantified parameters: distance moved **(A)**, freezing time **(B)**, number of entries in the center zone **(C)**, and the number of rearings **(D)**. Legend: *p < 0.05, **p < 0.01, ***p < 0.001, CTRL–control group icv injected with buffer and treated with vehicle (n = 8), CTRL + MEL–group icv injected with buffer and treated with melatonin (n = 8), STZ–group icv injected with streptozotocin and treated with vehicle (n = 7), STZ + MEL–group icv injected with streptozotocin and treated with melatonin (n = 6).

All animals showed unaltered locomotor activity and neophobia-induced anxiety when placed in the apparatus. Analyzing the activity of rats during a 5-min testing session, two-way ANOVA tests showed significant interaction between STZ injection and melatonin treatment [F (1, 25) = 7.552, *p* = 0.0110] for the distance travelled in the testing box. Post-hoc Tukey’s multiple comparison test revealed significant increase in the CTRL + MEL group compared to CTRL and STZ + MEL groups (Figure 2A). Accordingly, the freezing time of CTRL + MEL group was significantly reduced (Figure 2B). The number of entries and the time spent in the center zone of the apparatus were increased by the ICV-STZ injection, the intervention showing a significant main effect in the two-way ANOVA test (F (1, 24) = 4.947, *p* = 0.0358; F (1, 24) = 22.00, *p* < 0.0001). Interestingly, melatonin treatment did not influence these parameters (Figure 2C). Conversely, the vertical exploratory activity expressed as the number of rearing was increased by melatonin treatment [F (1, 25) = 10.73, *p* = 0.0031; Figure 2D], the CTRL + MEL group showed significant increase compared to CTRL and STZ + MEL groups. The self-grooming activity of the animals was also studied, but no effect of ICV-STZ injection or melatonin treatment was observed.

#### 3.1.2 NOR test

**FIGURE 3:**
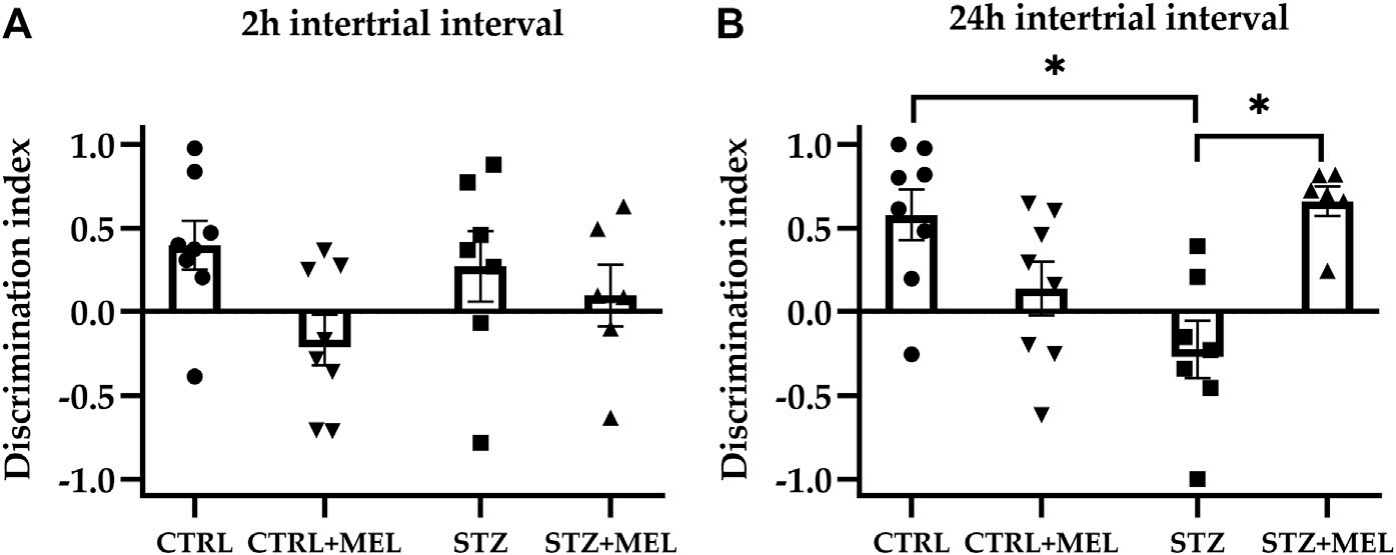
The discrimination index derived from novel object recognition task as a measure of recognition memory. Two intertrial intervals were tested: **(A)** 2-hrs and **(B)** 24-hrs. Data is expressed as mean ± SEM. Legend: *p < 0.05, CTRL–control group icv injected with buffer and treated with vehicle (n = 8), CTRL + MEL–group icv injected with buffer and treated with melatonin (n = 8), STZ–group icv injected with streptozotocin and treated with vehicle (n = 7), STZ + MEL–group icv injected with streptozotocin and treated with melatonin (n = 6).

In the familiarization phase, the two-way ANOVA analysis revealed no significant effect of ICV-STZ injection or melatonin treatment on the exploration of identical objects in the acquisition trial [F (1, 22) = 1.344, *p* = 0.2587; F (1, 22) = 1.577, *p* = 0.2223]. This means that there was no external influence on object preference. The first retention trial was performed after a 2-h intertrial interval, when a novel object replaced one of the familiar objects. One-way ANOVA revealed no significant differences for the DI values (Figure 3A) [F (3, 21) = 1.649, *p* = 0.21]. However, a high dispersion of the data was observed, so it was not possible to estimate the effect of melatonin treatment correctly. However, there was a significant effect of melatonin treatment on DI values observed during the second retention trial [F (3, 24) = 6.44, *P* = 0.002]. Post hoc Tukey’s multiple comparison test showed a significant decrease in DI following STZ injection (*p* = 0.005) when compared to the CTRL group. The other significant finding is that melatonin treatment administered after STZ injection increased the DI values significantly compared to the STZ group, up to the level of the CTRL group (Figure 3B).

#### 3.1.3 RAM test

**FIGURE 4:**
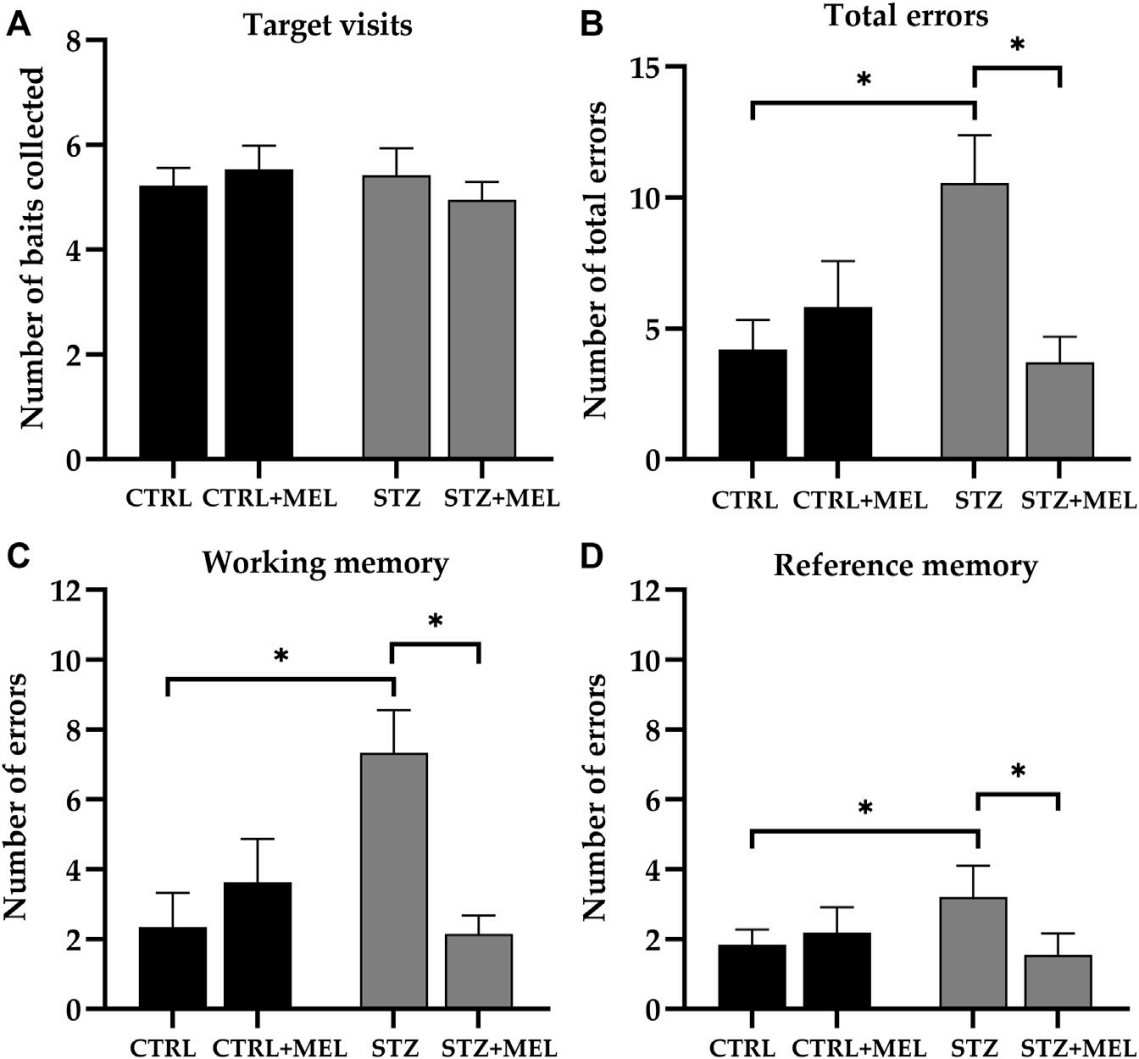
Radial arm maze test performance of melatonin treated rats underwent ICV-STZ injec-tion. Quantified parameters: target visits **(A)**, total errors **(B)**, working **(C)**, and reference memory errors **(D)**. Legend: *p < 0.05, CTRL–control group icv injected with buffer and treated with ve-hicle (n = 8), CTRL + MEL–group icv injected with buffer and treated with melatonin (n = 8), STZ–group icv injected with streptozotocin and treated with vehicle (n = 7), STZ + MEL–group icv in-jected with streptozotocin and treated with melatonin (n = 6).

For the evaluation of spatial learning and memory functions animals were tested on RAM. A high level of variability was observed, especially in the animals injected with ICV -STZ, during the 7-day learning period. Concerning the target visits, all groups performed similarly during the four test trials that comprised the test phase. This confirmed that all animals were capable of learning to complete the task and no differences were observed between the control and treated groups regarding the time needed to complete the task (*p* > 0.05). To evaluate the reference and working memory a three-way ANOVA analysis was performed using ICV-STZ injection, melatonin treatment, and trial number as main factors. The main effects of ICV-STZ injection and melatonin treatment were significant in the case of total errors [F (1, 92) = 3.954, *p* = 0.0497; F (1, 92) = 5.981, *p* = 0.0164, respectively] and working memory errors [F (1, 92) = 4.017, *p* = 0.0480; F (1, 92) = 4.952, *p* = 0.0285, respectively]. Moreover, an ICV-STZ × melatonin treatment interaction was also observed in the case of both parameters [F (1, 92) = 15.75, *p* = 0.0001; F (1, 92) = 13.59, *p* = 0.0004]. Interestingly, reference memory errors were significantly influenced by melatonin treatment [F (1, 92) = 5.027, *p* = 0.0274] only, but the interaction between ICV-STZ injection and melatonin treatment was also significant [F (1, 92) = 11.66, *p* = 0.0010]. The main effect of the trials and all interactions with the trials were not significant. So, the data was analyzed further using Tukey’s multiple comparison *post hoc* tests to find significant differences between the groups. As shown in Figure 4, the total number of errors [F (3, 23) = 7.290, *p* = 0.0013), Figure 4B], the number of reference memory errors [F (3, 23) = 6.543, *p* = 0.0023; Figure 4C], and the number of working memory errors [F (3, 23) = 5.381, *p* = 0.0059; Figure 4D] were significantly influenced by melatonin treatment. An increase in the number of errors in the case of the ICV-STZ group was observed, whereas melatonin treatment significantly decreased the STZ-induced alteration in cognitive performance.

### 3.2 Effects of melatonin supplementation on serum levels of TNF-α and MMP-9

**FIGURE 5:**
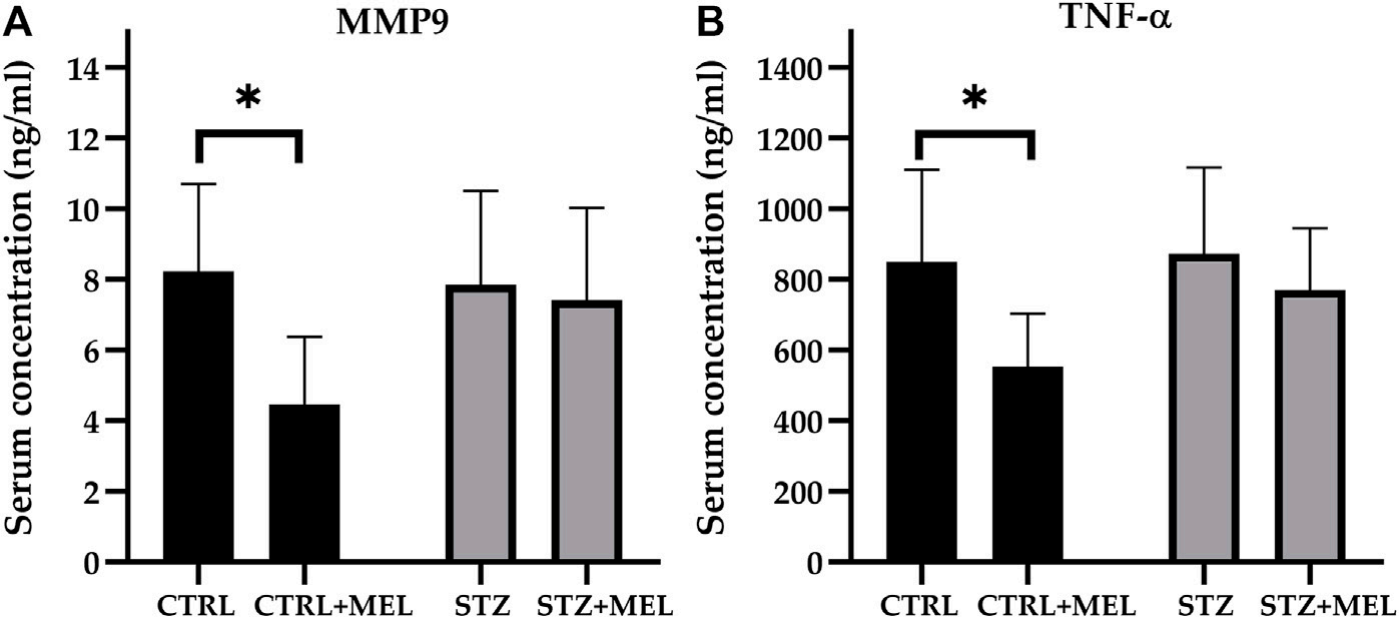
Serum levels of **(A)** MMP-9 and **(B)** TNF-α after melatonin treatment in ICV-STZ model of Alzheimer’s disease. Legend: *p < 0.05, CTRL–control group icv injected with buffer and treated with vehicle (n = 7), CTRL + MEL–group icv injected with buffer and treated with melatonin (n = 8), STZ–group icv injected with streptozotocin and treated with vehicle (n = 5), STZ + MEL–group icv injected with streptozotocin and treated with melatonin (n = 6).

In this study, the MMP-9 levels were determined using rat serum collected at the end of the experiment. Overall, melatonin treatment significantly influenced the MMP-9 levels as confirmed by the one-way ANOVA test [F (1, 21] = 4.706, *p* = 0.0417; Figure 5A). Post-hoc tests revealed that MMP-9 levels were reduced by melatonin treatment in the sham-operated control group, only a slight decrease being observed in the STZ-injected group. Similarly, TNF-α levels were significantly lower in melatonin-treated sham-operated animals than in sham-operated controls and STZ-injected animals [F (1, 21) = 5.385, *p* = 0.031]. The *post hoc* analysis with Sidak’s multiple comparisons tests revealed significant differences between groups (Figure 5B).

### 3.3 Effects of melatonin supplementation on cellular reorganization of the hippocampus

#### 3.3.1 NeuN immunoreactive cells

**FIGURE 6:**
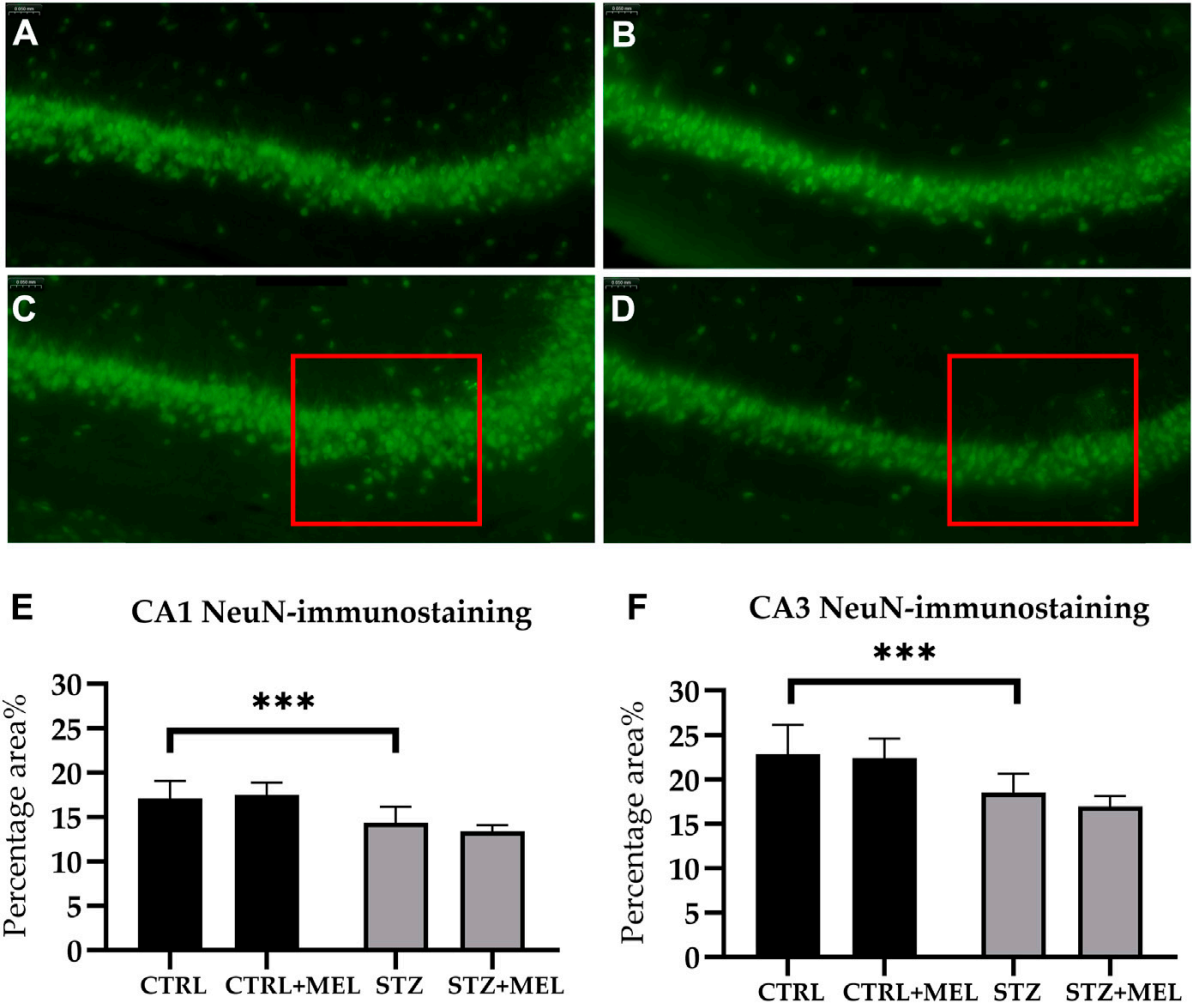
Effects of chronic melatonin (20 mg/kg body weight) treatment on the hippocampal re-organization induced by ICV-STZ in rats. Representative confocal microscopy images of the cornu Ammonis 3 (CA3) region **(A)** in the control group injected ICV with citrate buffer; **(B)** in the control group treated with melatonin; **(C)** in the ICV-STZ injected group; **(D)** in the ICV-STZ injected and melatonin treated group. The percentage area of neuron specific nuclear protein (NeuN) immunostaining (expressed as percentage of the area covered by positive signal compared to the total area of a region) revealed no effect of the melatonin treatment on STZ-induced cell loss in the **(E)** CA1 region and **(F)** CA3 region of the hippocampus. The magnification was ×20. The scale bar indicates 50 μm. Legend: ***p < 0.001, CTRL–control group icv injected with buffer and treated with vehicle (n = 10 hippocampi from n = 5 animals), CTRL + MEL–group icv injected with buffer and treated with melatonin (n = 16 hippocampi from n = 8 animals), STZ–group icv injected with streptozotocin and treated with vehicle (n = 20 hippocampi from n = 7 animals), STZ + MEL–group icv injected with streptozotocin and treated with melatonin (n = 12 hippocampi from n = 6 animals). The red rectangle indicates the region where significant modifications were observed.

Neuronal reorganization following STZ injection and melatonin treatment was examined in the hippocampus via neuronal nuclei antigen (NeuN) immunohistochemistry. NeuN-positive cells were observed in the pyramidal layer of the CA1 and CA3 hippocampal regions and the dentate gyrus’s granule cell layer. For STZ-injected animals, the pyramidal layer of the hippocampus showed a loose layout compared to those not injected with STZ (Figures 6A–D). Because of the limitations of the automated cell counting method used in this study (i.e., superposed cells or compact layers of cells could not be recognized and counted), the absolute number of NeuN cells could not be determined automatically. Instead, the percentage area% of the NeuN signal was evaluated in each CA1 and CA3 region. There were no differences between the groups in response to melatonin treatment (Figures 6E, F). However, STZ-injected groups showed decreased percentage area% of the NeuN signal in both CA1 and CA3 regions of the hippocampus [F (3, 54) = 15.95, *p* < 0.0001 and F (3, 54) = 25.46, *p* < 0.0001, respectively].

#### 3.3.2 GFAP immunoreactive cells

**FIGURE 7:**
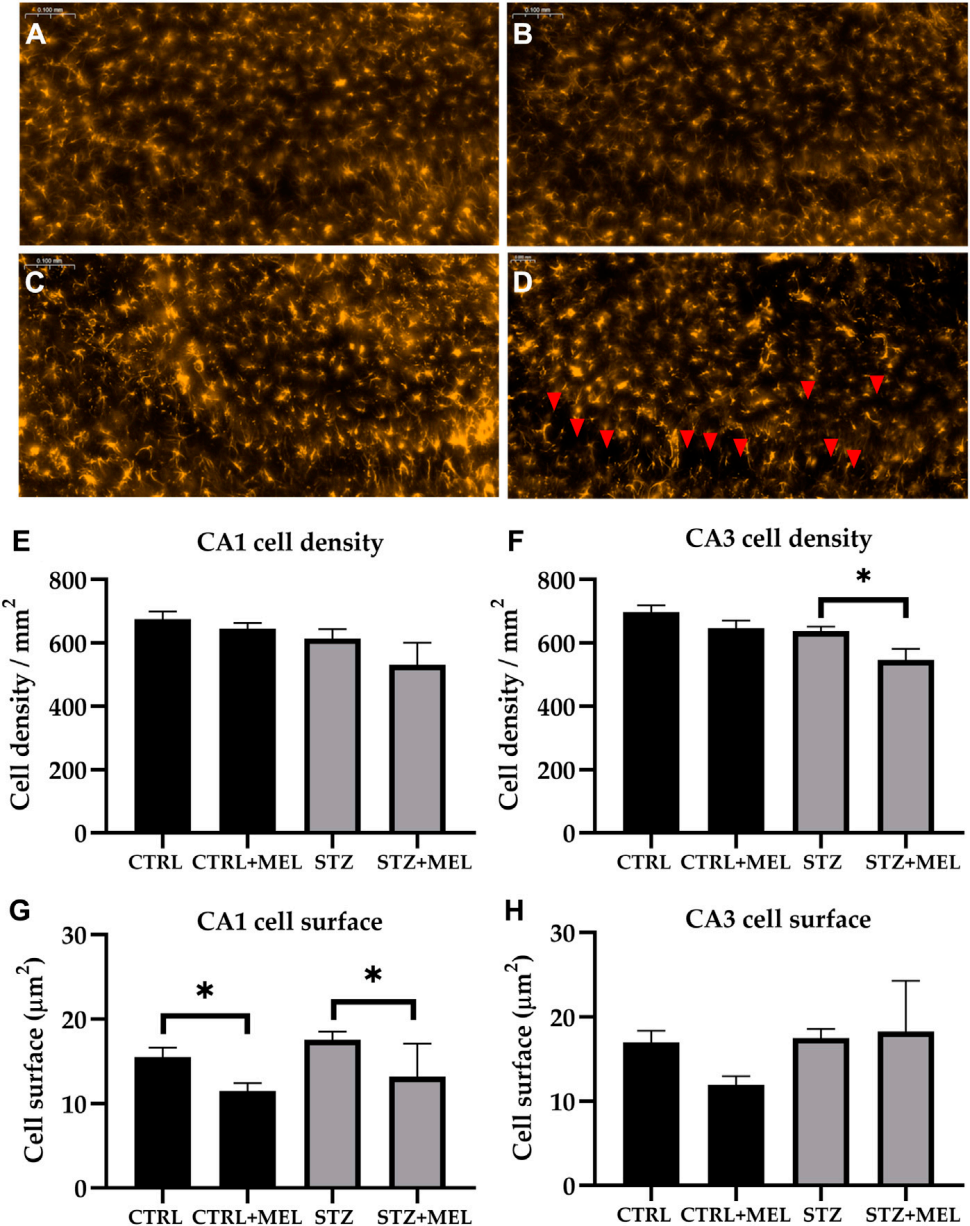
Effects of chronic melatonin (20 mg/kg body weight) treatment on the GFAP-positive astrocytes in the ICV-STZ sporadic model of Alzheimer’s disease in rats. Representative confocal microscopy images of the cornu Ammonis 3 (CA3) region **(A)** in the control group injected ICV with citrate buffer; **(B)** in the control group treated with melatonin; **(C)** in the ICV-STZ injected group; **(D)** in the ICV-STZ injected and melatonin treated group. The magnification was ×20. The scale bar indicates 50 μm. The astrocyte density (expressed as cell/mm^2^) revealed no effect of the melatonin treatment in the CA1 region **(E)** but it did reduce astrocyte density in the CA3 region **(F)** of the hippocampus. The astrocyte dimension (expressed as mean cell surface in μm^2^) showed significant decrease in both melatonin treated groups in the CA1 region **(G)**, however, the modifications did not reach the limit of significance in the CA3 region **(H)**. Data in column graphs are expressed as mean ± SEM (n = 5–8).; Legend: *p < 0.05, CTRL–control group icv injected with buffer and treated with vehicle (n = 10 hippocampi from n = 5 animals), CTRL + MEL–group icv injected with buffer and treated with melatonin (n = 16 hippocampi from n = 8 animals), STZ–group icv injected with streptozotocin and treated with vehicle (n = 20 hippocampi from n = 7 animals), STZ + MEL–group icv injected with streptozotocin and treated with melatonin (n = 12 hippocampi from n = 6 animals). Red arrowheads indicate the regions where significant differences were observed.

Astrogliosis associated with STZ induced neuroinflammation was studied in the hippocampus by glial fibrillary acidic protein (GFAP) immunohistochemistry (Figures 7A–D). The number of astrocytes was counted in the CA1 and CA3 regions of the hippocampus and the cell density was calculated. On the other hand, the mean astrocyte cell size was determined. STZ-injected animals showed no difference in the density and cell surface of GFAP-labeled astrocytes compared to CTRL group. However, melatonin treatment significantly reduced the cell density in the CA3, and the cell surface in the CA1 region (Figures 7E–H).

#### 3.3.3 IBA-1 immunoreactive cells

**FIGURE 8:**
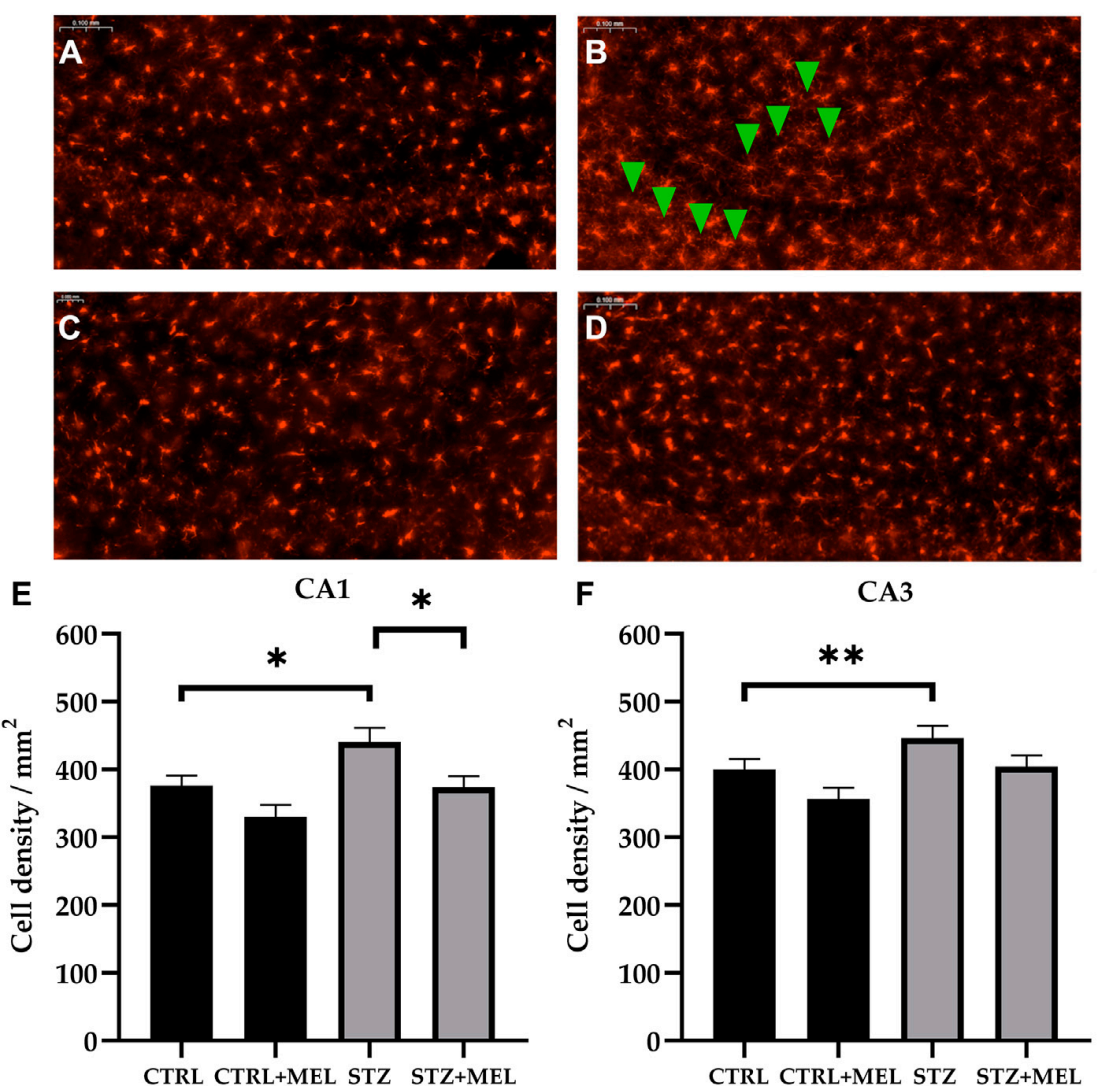
Effects of chronic melatonin (20 mg/kg body weight) treatment on the IBA-1-positive microglia in the ICV-STZ sporadic model of Alzheimer’s disease in rats. Representative confocal microscopy images of the cornu Ammonis 3 (CA3) region **(A)** in the control group injected ICV with citrate buffer; **(B)** in the control group treated with melatonin; **(C)** in the ICV-STZ injected group; **(D)** in the ICV-STZ injected and melatonin treated group. The magnification was ×20. The scale bar indicates 50 μm. The microglia density (expressed as cell/mm^2^) showed significant increase due to ICV-STZ injection in both CA1 and CA3 regions and melatonin treatment reduced this increase in the CA1 region **(E)**, however it did not reach significance limit in the CA3 region **(F)** of the hippocampus. Data in column graphs are expressed as mean ± SEM (n = 5–8).; Legend: *p < 0.05, **p < 0.01, CTRL–control group icv injected with buffer and treated with vehicle (n = 10 hippocampi from n = 5 animals), CTRL + MEL–group icv injected with buffer and treated with melatonin (n = 16 hippocampi from n = 8 animals), STZ–group icv injected with streptozotocin and treated with vehicle (n = 20 hippocampi from n = 7 animals), STZ + MEL–group icv injected with streptozotocin and treated with melatonin (n = 12 hippocampi from n = 6 animals). Green arrowheads indicate the regions where significant differences were observed.

Alterations involving microglia were studied in the CA1 and CA3 regions of the hippocampus by ionized calcium-binding adapter molecule 1 (IBA-1) immunohistochemistry. Microglia cell density was significantly different between treatment groups (Figures 8A–D). STZ injection increased the number of IBA-1 positive microglia compared to controls both in the CA1 (440.8 ± 20.8 vs. 376.1 ± 15 cell/mm^2^) and CA3 subregions (446 ± 18.4 vs. 400.1 ± 15.8 cell/mm^2^). However, melatonin treatment reduced it in the CA1 (373.5 ± 16.6 cell/mm^2^) and CA3 (404.2 ± 16.5 cell/mm^2^) regions of the hippocampus to a comparable level to controls (*p* < 0.05, Figures 8E, F).

## 4 Discussion

Alzheimer’s disease and dementia are associated with a decrease in melatonin production (Bubenik and Konturek, 2011; Nous et al., 2021) and an age-dependent decline of melatonin production was also demonstrated in rat models of AD, however, supplementation can restore the normal levels in serum and pineal gland (Rudnitskaya et al., 2015). During the last few years, melatonin has been recognized as a promising tool for treating conditions with inflammatory origins (Favero et al., 2017). It has also been recognized as a neuroprotective agent (Tozihi et al., 2023). Melatonin’s beneficial effects have already been reported in other AD models (Cheng et al., 2006) and traumatic brain injury (Ikram et al., 2021). Melatonin exerted antiapoptotic activity (Wang, 2009), decreased tau hyperphosphorylation (Das et al., 2020), stimulated neurotrophic factors (Miranda-Riestra et al., 2022), and reduced neuroinflammation by affecting microglia and astrocytes (Hardeland et al., 2015; Hardeland, 2021; Zhou et al., 2021). This study was conducted to investigate the potential efficacy of subchronic melatonin treatment in alleviating the cognitive decline and neurodegeneration induced by ICV injected STZ. The results presented here showed that melatonin treatment improved cognitive performance of rats in both the novel object recognition and radial arm maze tests, reduced the levels of TNF-α and MMP-9 in the serum, and led to a reduction in astrogliosis and microglia cell density in the hippocampus.

The progression of AD has previously been linked to neuroinflammation, free radical formation and mitochondrial dysfunction with consequent neuronal loss, as well as to increased glutamatergic signaling (Rosales-Corral et al., 2012). Based on this hypothesis, several animal models were used to elucidate the pathophysiological features and behavioral manifestation of neurodegeneration. STZ, a glucosamine-nitrosourea derivative, causes diabetes by targeting GLUT-2 transporters in the pancreas when administered intraperitoneally. But injected intracerebroventricularly STZ induces lesions that resemble human AD including metabolic, neuropathological, and behavioral disturbances (Grieb, 2016; Kamat et al., 2016; Ravelli et al., 2017). Although the exact mechanisms are not known, this animal model became popular in the last decade because it can provide an insight into the temporal dynamics and the nature of the pathophysiological processes (Homolak et al., 2021). Injection of STZ into the brain results in neuroinflammation, oxidative stress, impaired orientation and memory, reduced insulin receptor function, excitotoxicity, increased tau phosphorylation, amyloid accumulation, and synaptotoxicity. The presence of neuroinflammation was detected 1 week after the administration of 3 mg/kg of STZ, and information processing, episodic, working, and reference memory problems were noted (Kraska et al., 2012). Furthermore, this model can be used to identify and observe cellular reorganization of key brain regions, since activation of microglia and astrocytes is affected by inflammatory cytokines, as well as oxidative stress (Mishra et al., 2018). In this study, STZ-injected rats showed an increased number of microglia and astrocytes in both CA1 and CA3 regions. Melatonin treatment reduced the number of microglia and the surface area of astrocytes.

Cognitive impairment is a common feature of several pathological conditions, including Alzheimer’s disease and traumatic brain injury. Melatonin is an endogenous hormone that plays a role in circadian rhythm regulation, sleep-wake cycles, and neuroprotection. Recent studies have also suggested that melatonin may have cognitive enhancing effects, although the underlying mechanisms are not fully understood. Recently, Andrade et al. (2023) found that long-term melatonin treatment reversed the cognitive impairment in the Y-maze induced by ICV-STZ; however, it did not influence the performance of rats in the object location test. These differences could be associated with the different effects of melatonin on the involved brain regions. Or melatonin may have a dose-dependent effect on different learning tasks. For example, Madhu et al. (2021) conducted a dose-response study assessing the cognitive improvement caused by melatonin in a persistent cognitive and mood dysfunction model in rats. Based on their findings, melatonin’s effects on cognition were dose-dependent—in low doses, recognition memory is affected, while in higher doses, more complex cognitive functions are improved. The novel object recognition and radial arm maze tests are widely used behavioral paradigms to evaluate cognitive function in rodents (Antunes and Biala, 2012). The novel object recognition test assesses recognition memory, while the radial arm maze test evaluates spatial memory and learning. Our results demonstrate that melatonin treatment improves performance in both tests. As a possible explanation for cognitive enhancement, it should be noted that melatonin increased cholinergic transmission by inhibiting AChE in the neocortex and hippocampal regions (Rosales-Corral et al., 2012). This could be a similar mechanism to the existing AChE inhibiting drugs, such as rivastigmine, galantamine or donepezil. Nonetheless, this study provides further evidence for a potential therapeutic application of melatonin in the treatment of cognitive impairment.

TNF-α and MMP-9 are pro-inflammatory cytokines and proteases, respectively, which have been implicated in the pathophysiology of cognitive impairment. Elevated levels of these biomarkers have been observed in various pathological conditions, including Alzheimer’s disease and traumatic brain injury. The role of TNF -α in Alzheimer’s disease has been recently reviewed by Plantone et al. (2023). In astrocyte cell culture TNF-α induced amyloidogenic processes including Aβ production (Zhao et al., 2011). In clinical setting, it has been demonstrated that peripheral TNF-α elevation enhances brain pro-inflammatory cytokine expression and Alzheimer’s disease patients exhibit high level of serum TNF-α concentrations (Culjak et al., 2021). Moreover, some evidence suggests that serum TNF-α concentrations are negatively correlated with cognitive function (Kim et al., 2017). Thus, any intervention that decreases serum TNF-α levels might be protective for brain health and cognitive status (Park et al., 2019; Li et al., 2022). On the other hand, an elevated MMP-9 level has also been reported in Alzheimer’s disease and a variety of neurological and inflammatory diseases (Hernandes-Alejandro et al., 2020). However, Ringland et al. (2021) showed that MMP-9 inhibition alone did not improve spatial learning and memory in mice, as measured by the radial arm water maze. As described earlier, TNF-α levels increase to a maximum level after 6 h following STZ-injection and remain elevated for a 1-week period (Souza et al., 2017). This is in line with the results obtained by Rai et al. (2014), suggesting that neuroinflammation and glial activation induced by STZ-injection occur earlier (7–9 days after STZ-injection) than cognitive impairment, which takes 14–16 days to become visible. However, there is no evidence whether the chronic stage is characterized by elevated TNF-α and MMP-9 levels. In the current work, no difference was observed between sham-operated and STZ-injected animals at the end of the experiment, 37-days after STZ-injection. On the other hand, melatonin treatment significantly reduced the levels of TNF-α and MMP-9 in the serum of sham-operated animals. However, the reduction did not achieve statistical significance in STZ-injected animals Further experiments with additional treatment groups are needed to clarify the effects of melatonin treatment on TNF-α and MMP-9 levels in the chronic phase.

Astrogliosis, a hallmark of Alzheimer’s disease, refers to the hypertrophy and hyperplasia of astrocytes in response to various insults, including neuroinflammation. Astrocytes play a key role in the maintenance of the central nervous system and are involved in a variety of physiological and pathological processes. However, excessive astrogliosis can lead to the formation of a glial scar, which can impede axonal regeneration and functional recovery following injury. STZ injection was demonstrated to increase astrocyte density in some brain regions, but not in the hippocampus (Humphrey et al., 2023). This is in accordance with the results of the current study, where no increase was observed due to STZ-injection. On the other hand, the current work showed that melatonin decreased astrocyte density in the CA3 at a dose of 20 mg/kg and reduced the astrocyte surface in the CA1 (i.e., reducing astrocyte ramifications without decreasing their number). Although, Andrade et al. found no influence of melatonin treatment on the GFAP protein expression in the hippocampus at a dose of 10 mg/kg melatonin (Andrade et al., 2023), it might exert direct inhibition on cell proliferation at higher doses (Estaras et al., 2023).

Similarly, microglia, the resident immune cells of the brain, play an important role in the regulation of neuroinflammation. In response to injury or infection, microglia become activated and release pro-inflammatory cytokines and chemokines. While this response is necessary for the clearance of pathogens and damaged cells, excessive microglial activation can lead to chronic inflammation and damage to healthy tissue. As microglia play a critical role in β-amyloid induced synapse loss and cognitive decline in AD, they might represent a potential therapeutic approach in AD treatment (Miao et al., 2023). Melatonin was shown to reduce microglial activation both in the scopolamine induced amnesia rodent model (Muhammad et al., 2019) and the kainic-acid model of hippocampal neurodegeneration and oxidative stress (Chung and Han, 2003). Nasiri et al. (2019) described that melatonin pretreated stem cells therapy significantly reduced IBA-1 positive glial cells number in a rat model of AD. The results of this study provide further support that melatonin treatment may reduce microglia cell numbers and activation in the injured brain. Recently, one of the possible mechanisms by which melatonin could ameliorate hippocampal damage, namely the inhibition of the Cathepsin B/nucleotide-binding oligomerization domain-like receptor pyrin domain-containing 3 (NLRP3) signaling pathway, was revealed (Gao et al., 2024). To note, β-amyloid plaques and tau aggregates stimulate microglia and astrocytes, which trigger the chronic neuroinflammatory response, neuronal death and pyroptosis through the activation of the intracellular NLRP3 inflammasome (Liang et al., 2022). Therefore, the potential inhibitory effect of melatonin on inflammatory cytokines, astrogliosis and microglial function along with its cognitive enhancing activity might confer an crucial role for melatonin in treating AD.

Finally, the current study has some limitations which should be acknowledged (Livingston et al., 2017): the experiment was performed using male animals only, whereas AD affects more women than men and sex differences could be found in disease progression and phenotype (Estrada and Contreras, 2019); the effects of melatonin described here were not supported by mechanistic investigations, the association between improved cognitive performance and decreased activation of microglia needs further investigations.

## 5 Conclusion

Melatonin treatment improved the cognitive performance of rats and reduced the activation of microglia in both CA1 and CA3 regions of the hippocampus. These findings support the existing evidence for melatonin as a potential therapeutic option in neurodegenerative diseases, such as Alzheimer’s disease. Further research is needed to confirm these findings and probe the underlying mechanisms.

## Data Availability

The raw data supporting the conclusions of this article will be made available by the authors, without undue reservation.
